# On agent-based modeling and computational social science

**DOI:** 10.3389/fpsyg.2014.00668

**Published:** 2014-07-14

**Authors:** Rosaria Conte, Mario Paolucci

**Affiliations:** Laboratory of Agent Based Simulation, Institute of Cognitive Science and Technologies, CNRRome, Italy

**Keywords:** agent-based modeling, computational social science, agent-based simulation, interdisciplinarity, multi-realizability, model building

## Abstract

In the first part of the paper, the field of agent-based modeling (ABM) is discussed focusing on the role of generative theories, aiming at explaining phenomena by growing them. After a brief analysis of the major strengths of the field some crucial weaknesses are analyzed. In particular, the generative power of ABM is found to have been underexploited, as the pressure for simple recipes has prevailed and shadowed the application of rich cognitive models. In the second part of the paper, the renewal of interest for Computational Social Science (CSS) is focused upon, and several of its variants, such as deductive, generative, and complex CSS, are identified and described. In the concluding remarks, an interdisciplinary variant, which takes after ABM, reconciling it with the quantitative one, is proposed as a fundamental requirement for a new program of the CSS.

## 1. Introduction

The two decades around the turn of the millennium have seen the rapid advent, and perhaps the premature decline, of a paradigmatic shift in science, represented by agent-based modeling (ABM) and simulation. In this section, after shortly defining what we mean with ABM, we present a short account of its history.

### 1.1. What agent-based modeling is

What is meant by Agent Based Modeling? Often, this is defined in opposition to Equation-Based Modeling (see for example Dyke Parunak et al., 1998; Cecconi et al., 2010). More specifically, ABM arises at the intersection between agent theory, systems, and architectures, on one hand, and the social sciences, on the other hand. Agents are usually defined (see Conte, 2009) as autonomous systems that operate transitions between states of the world, based on mechanisms and representations somehow incorporated into them.

Under this general definition, the field of agents shows a tremendous variability. Agents vary indeed on several dimensions, which include whether and to what extent they are autonomous, self-interested, sociable, and capable to learn from experience and/or observation. Agents also differ in their level of complexity: according to a classic distinction introduced by Wooldridge and Jennings in their influential work (Wooldridge and Jennings, 1995), agents in a “strong” sense are capable to manipulate and reason upon mental representations; otherwise they are considered agents in a “weak” sense. Another important distinction concerns the way in which mental representations are incorporated: symbolic representations allow an agent to mentally manipulate them in order to reason, plan, take decision, communicate. Sub-symbolic representations are unaware, implicit, based for example on network-like configurations representing the structure of relationships among neurons in cerebral areas, and not liable to purposive manipulation on the side of the agent. Finally, agents vary according to the philosophical or meta-theoretical view their description is based upon. One example is the attempt to model agents on the basis of a personal utility function, on which much work on agents has been done over the past 30–40 years or so, and that has also been criticized as for its micro plausibility (Antunes and Coelho, 2004).

The practice of ABM however did represent a substantial under-exploitation of such wide spectrum of possibilities. De facto, much of the agent models worked out and simulated are totally *ad-hoc*, based on very simple local rules (Epstein, 2006), more or less arbitrarily implemented on a program running on a computer (Gilbert and Troitzsch, 2005). When the program is run, macroscopic effects of the local rules can be observed on the screen, and then be stored, analyzed and possibly visualized in search for emergent phenomena. We will return to the problem of *ad-hoc* rules in section 2.3 below. Such a practice of modeling lends itself well to observe and experiment upon multi-agent worlds or agent societies. These are meant to either reproduce some real-world setting or phenomenon [a typical example is the Anasazi culture simulation (Axtell et al., 2002)], or to build up and observe would-be worlds (Casti, 1997). Such models allow novel theories about abstract social phenomena to be formulated, operationalized, and tested. Examples of this application of ABM abound and are among the best cited works so far worked out in this field.

### 1.2. Agent-based modeling in a historical perspective

Conference proceedings, dedicated to the new methodology of ABM and its multiple applications within the social and behavioral areas of science, started to appear in Europe since the early nineties (Gilbert and Doran, 1994; Gilbert and Conte, 1995; Conte et al., 1997). Agent-based models of social phenomena trace back to as early as 1971, when the famous (Schelling, 1971) model of segregation was published in the Journal of Mathematical Sociology. In 2002, the field obtained a major institutional acknowledgment, when the proceedings of a Sackler Colloqium of the National Academy of Sciences, under the title “Adaptive Agents, Intelligence, and Emergent Human Organization: Capturing Complexity through Agent-Based Modeling,” held in October 2001, were published on PNAS (Bonabeau, 2002). In that circumstance, ABM was proclaimed as the leading field—we might say the flagship to use a trendy tag - in the renewal of the social, behavioral, and complexity science, which was expected to take place in the years to come. Consecrated by the US scientific institutions, the field was already intensely practiced also, if not primarily, in Europe, where ABM had given rise to a new journal, the Journal of Artificial Societies and Social Simulation (JASSS, founded in 1998), to the first scientific association (ESSA, The European Social Simulation Association, created in 2003), and was at the center of a variety of promotional activities. Soon enough its range of influence extended beyond the two sides of the Atlantic, reaching out to the Pacific area, and giving rise to the PAAA association. At the same time, the NAACSOS association was founded in the US. After some years of fruitful competition, the associations joined in the first World Conference on Social Simulation held in Kyoto in 2006.

At the end of the first decade, however, the ABM leadership seems to be challenged if not decisively weakened by the (re)appearance of a more sober, more encompassing, and less innovative tag, that of Computational Social Science (CSS), of which ABM is a component (see Bankes et al., 2002) for an early insight), and which now candidates itself to replace ABM in the same leading position for the next decade. Evidence of a change of leadership and of a possible coming era for bare CSS, rather than for the more inspiring Generative Social Science proposed by Epstein in 2006, can easily be found in some position papers (e.g., Lazer et al., 2009; Cioffi-Revilla, 2010), books recently appeared (Gilbert, 2010), a new regional association—the CSS Society of the Americas, born on the ashes of the short-lived NAACSOS, and the relative conference held in 2011—and, finally, the objectives of the unsuccessful but groundbreaking EU FET flagship pilot FuturIct (www.futurict.eu).

If history is instructive, the study of signaling is fun. In the era of information overflow, distributed content production, collaborative filtering, crowd sourcing, and so on, emblems are decisive. Tags have a far-reaching but short life. Under the tyranny of PageRank, contents compete in terms of lookups, and these most certainly depend on familiarity, and possibly also on tags appeal. Science makes no difference. It is somewhat surprising when a paradigm shift is signaled by a flat combination of two traditional scientific areas: social sciences and computational science. What is the meaning conveyed by this signal? Does the new label correspond to a new paradigm shift in the social and behavioral sciences, or does it simply meet a kind of marketing need for periodical renewal of names?

This paper presents an attempt to weigh up the impact of ABM and answer the question whether this field is undergoing or not a real decline; whether or not his replacement was timely, necessary, and effective. Next, some current variants of CSS will be compared. Finally, some important requirements for achieving real progresses in the computational study of social phenomena will be identified and discussed.

## 2. Agent-based modeling: a balance

Rather than a detailed survey of ABM (for a good example, see Helbing and Balietti, 2011a) this paper presents an attempt to draw a balance of this field, pointing to its main weaknesses and strengths.

### 2.1. Strength of agent-based modeling

One may wonder what ABM is good for and what are its major strong points. The tricky questions as to when ABM is really needed, whether agent-based models can or cannot be converted into an analytical, equation-based model and to what extent this can be done has been debated at length elsewhere (see for example Epstein, 2006; Cecconi et al., 2010). Nonetheless, ABM remains the only known approach apt to model and reproduce sets of heterogeneous agents interacting and communicating in different ways.

Of course, ABM can only provide a sufficient explanation of the phenomenon of interest, not a necessary one. This feature, which (Epstein, 2006) extensively clarifies and discusses, is also known as *multi-realizability* (Sawyer, 2005), and it is an outstanding property of multilevel systems. A macro-level phenomenon in whatever domain social, natural, mental, etc. of reality, is multi-realizable when it can be implemented in different ways on the lower levels. Inevitably, ABM generates the higher-level effect by following one of the possible generating paths. Even if as many models as generating paths were actually implemented, it would still be difficult, if not impossible, to assess which one among them is effectively implemented in the real world. But, interestingly, this is true also of the target phenomena: an organization can perform its mission independently of the internal structure (consider as an example a project-based structure against a functional one). Social conformity is achieved through a variety of internal mechanisms, e.g., imitation or norm compliance. It is still unclear how disapproval works as a sanction, whether it affects people's decision-making because it activates an expected associated material punishment, or violates the goal of a good (self) esteem. Actually, multi-realizability is a property not only of ABM but also of the real world. In this sense, multi-realizability differs from the more general issue of model underdetermination, as it connects it directly with possible generative paths in reality, an analogy that makes ABM particularly apt to study the equivalence, or possible lack thereof, of structure and mechanisms inside intermediate levels, in the sense of the examples above.

To implement sets of heterogeneous agents in interaction brings about a series of second order advantages: agent societies are (1) operational platforms where theories get converted into falsifiable hypotheses; (2) experimental laboratories where theories get gradually and thoroughly controlled; (3) multilevel worlds where the level of individual units, the agent, is clearly distinct from the macro-level, the system level and unforeseen effects and emergent properties of interaction can be observed.

In short, ABM is an *in nuce* society, which unfolds and actualizes when the model is implemented on a computer program and this runs. In some cases, the effects observed in the computer could not be predicted while modeling and implementing the single units, the individual agents. Hence, the effects of such behavioral units on the whole agent society or parts of it can be observed and investigated. Otherwise stated, ABM allows the interplay between different levels of a social system to be modeled and observed. As shall be seen later, this important property of real-world societies has been insufficiently exploited. The main dynamic investigated by ABM is the way-up of the interaction among the micro and the macro-level. The complementary process, the way-down from the macro-level to the micro-level, has been poorly explored. Closing the loop, however, may require a high level of ABM complexity. Theory-driven, non-*ad-hoc* models of phenomena generated by intelligent behavior may be relatively difficult to calibrate (Heckbert et al., 2010). Difficulties usually increase with the model's level of scale and the number of parameters. One may perceive a trade-off between vertical scaling, i.e., agent complexity, and horizontal scaling, i.e., scenario complexity. Such a trade-off is probably one of the keys for ABM development and leads us straightforward to one of the weak points in the field.

### 2.2. Weakness of agent-based modeling

Some problems and difficulties in the field of ABM and simulation have been perceived from within the scientific community since long, while others have only recently come to our attention. Since the field's early days, a serious concern of Agent Based modelers and simulators is how to design large-scale agent-based simulations. In its initial applications, agent-based models did not care much about the problem of scale, as they were applied to observe the emergence of patterns from interaction at the microscopic level in artificial scenarios sharing some crucial features of the real-world, but not really aimed to reproduce its details. As soon as the potential of agent-based models became apparent—revealing a great occasion for observing and manipulating *in silico* models of target phenomena in order to acquire a better control, and possibly to optimize intervention—upgrading their level of scale of several orders of magnitude proved necessary. You cannot optimize a system of traffic if you do not manipulate parameters in populations of several millions of agents.

Under the pressure of complex systems science, which is gaining ground in the study of social phenomena (Helbing and Balietti, 2011b), agent-based simulation is increasingly expected to meet a further, and connected, important requirement, i.e., to be fed by massive data in real-time. To answer the problems of scale and real-time simulation, a variety of ICT solutions (parallel and supercomputing infrastructures) are being designed and tested. To deal with this challenge, agent-based simulations were bent to applications needs, such as policy modeling and traffic optimization (Grether et al., 2010), distributed communication over the Internet (Chen, 2009), electricity market (Guerci et al., 2010), financial crisis (Sornette, 2003), epidemics (Pastor-Satorras and Vespignani, 2001). This is not the forum for discussing sophisticated technical solutions (but for a review of techniques to that purpose, the reader might be referred to Paolucci et al., 2013) to the problem of making ABM more apt to the requirements of BigData science. We will instead touch briefly on the question of model equivalence across disciplines and applications.

#### 2.2.1. Equivalence of models

Unlike laws of nature, Agent Based models of socio-economic phenomena are countless and not always consistent (see Alfi et al., 2009). Think of the various heuristics and rules of thumb applied in defining microscopic rules for ABM. Most of them generate results at the macroscopic level, which are applied more or less the same narratives or metaphors. Hence, cooperation in Prisoner's Dilemma is found to emerge from a set of heterogeneous strategies, from TIT-FOR-TAT (Axelrod, 1997) to strong reciprocity (Boyd et al., 2003), from image-scoring (Nowak and Sigmund, 1998) to reputation-building (Pinyol et al., 2012), and finally group selection (Di Tosto at al., 2007); social control is found to emerge from ostracism (Xenitidou and Elsenbroich, 2010), but also from partner selection, and finally from gossip (Giardini and Conte, 2012); norms emerge from punishment (Galán and Izquierdo, 2005), which in turn is but a TIT-FOR-TAT strategy, but can also emerge from conditioned preferences (Bicchieri, 2006), and from habituation (Epstein, 2008). Is models' equivalence a major shortcoming of the field, or something social scientists can put up with? What does it depend upon? Is it a necessary or a contingent feature of ABM?

We believe the variety of equivalent agent models in part depends on a property inherent to multi-level systems as complex social systems are. The property in question is the multi-realizability that we have mentioned above. In part, we believe it to be a consequence of the shaky foundations, the poor theoretical justification at the basis of many agent models. This is not equal to finding poorly realistic the model of agent often proposed by current modelers, and asking to improve it toward psychological, cognitive, or sociological plausibility - toward a *seemingly human* agent. What is wrong, in our view, is the procedure for model building and the role of behavioral rules. Let us examine both points with some detail in the next two sections.

### 2.3. The ABM recipe for model building

A consensus seems to have emerged in ABM on a minimality procedure; that is, models are built by setting up the rules that are minimally required to obtain the macroscopic effect to be described. While minimality might sound obviously inspired by the success of hard sciences, the substantial failure to apply such a minimality procedure to social science is testified by centuries of failed attempts, starting from what had been announced as “Social Physics” in the seventeenth century (for an historical perspective, see Ball, 2002). The reasons for consensus on minimality might be better described with the tools of the sociology of science than rooted in the search of theoretically sound and scientific advances. Indeed, the ABM community, being still relatively small, is subject to issues of disciplinary recognition, with the consequent pressure to publish in a limited number of outlets; and it might still be looking for the right dimension of the contribution - the ideal paper size, as measured in effort invested and soundness of results, could be very different from the “correct” paper size in terms of publication chances. This discrepancy causes a motivational pressure toward minimal (and publishable) models, and hampers research in the much more interesting issue, why minimality seems to fail in the social sciences. We will get back to our intuition on this matter in the conclusions.

Under the rule of minimality, model building is operated (1) a posteriori, based on backward engineering from the effects obtained to the generating rules; (2) *ad-hoc*, so that rules are suggested by the specific results to be obtained; (3) in a rule-oriented rather than agent-oriented approach: what is achieved is a set of rules, rather than an agent view; (4) inspired by the minimal-conditions logic: modeling consists of finding out a set of microscopic rules minimally required to reproduce a given phenomenon of interest. The minimal approach, thus, strongly reduces the validity of ABMs on two separate accounts. On the one hand, theory-based, agent models are implausible caricatures of agent as prescribed by the rationality theory, with a touch of psychological realism in the best possible case. On the other hand, when agent models are not derived from any pre-existing agent theory or vision, whether computational or not, but only by the behavior they are expected to generate (Epstein, 2006), agent models become arbitrary, poorly comparable, competent in highly specific domains of knowledge and disarmingly inapt in any other. It should not come as a surprise if, as a result, a myriad of rather inconsistent agent-based models have been produced over the past 20–30 years or so. Is it possible to find an escape between implausible models and arbitrary ones, or between *ad-hoc* rules and useless ones? Options exist, but are poorly exploited. Why?

#### 2.3.1. From cognitive models…

One such option is represented by cognitive agent models, which exist since the late nineties. Their wide range of influence is shown by the popularity of BDI architectures (about 32,700 “BDI agents” cites on Google Scholar retrieved on March, 18th 2013) within and beyond the field of agent systems and theories. Simulation of social phenomena with BDI based models also abound in the literature (about 7060 “BDI social simulation” cites on Google Scholar on March, 18th 2013), and usable platforms to implement them are under consolidation, from Jason (Bordini et al., 2007) to Netlogo extensions (Sakellariou et al., 2008). However, works with this approach receive attention mostly from the computer science community, and are rarely published in main social scientific journals.

Although the rich cognitive models tag appeared since the early nineties (for a recent example see Dignum et al., 2010), the amount of models inspired from it remains negligible. Sub-symbolic systems and neural nets did not make much better. Although neural nets and social simulation fare better, relative publications again do not appear in major social scientific journals. Why are cognitive theories on agency, whether symbolic or subsymbolic, so poorly applied in ABM? In part, there are problems of inner validity and calibration. While it is difficult to control the inner validity a complex agent-based model (Cioffi-Revilla, 2002; Windrum et al., 2007), to calibrate it and manipulate parameters values so to reflect a real-world system is hard. To gather data on which the agent model is based upon takes more time and more complex empirical methodology. Therefore, the utility of a complex agent model to simulate the real-world system (i.e., showing that the model's results match the real-world data) is questionable (Crooks et al., 2008). Undoubtedly, these difficulties reduce the interest of cognitively grounded models simulators, although the latter's foundations are much firmer than those of most of the models used. The lesson one might draw is that, like it or not, scientific developments are often due to practical utility more than theoretical soundness. However, the little success of cognitively grounded agent-based models is also due to other factors.

First, unlike other theory-grounded agent models, for example the rational models, cognitive models are not prescriptive. Whereas the theory of rationality is a theory of action, cognitive modeling provides theories of the agent. Hence, the rational agent model fits only apparently better the objectives of ABM and simulation, but it does so only because it allows the modeler to get rid of the tricky part of the modeling, that is, how agents form the goals, the motivations, the preferences, that will be implied in the decisions.

#### 2.3.2. .. to generative models.

Secondly, cognitive modeling is a truly generative theory of behavior, accounting for behavior in terms of the mechanisms that are supposed to operate while producing it. A generative explanation of an observed social phenomenon consists of describing it in terms of the external (environmental and social) and internal (behavioral) mechanisms that generate them, rather than by inferring causes from observed co-variations. This is a vital property of explanation, which cannot easily be realized otherwise. When describing agent behavior by means of other formalisms (logic-based or numeric), we describe behavior from the outside, as perceived by an observer, but do not describe the way it is generated. ABM explains behavior from within, in terms of the mechanisms that are supposed to have generated it, that is, the mechanisms that operate in the agent when s/he behaves one way or another.

Of course, behavior can be explained otherwise. For example, the flight of hawks is wonderfully explained by the mathematical property of logarithmic spiral, such that any tangent from the center of the spiral yields an angle of the same width. Thanks to this property, hawks can keep their preys always in their aim while describing a spiral before pouncing on them. But this explanation is not generative, in the sense that it does not tell us what are the internal mechanisms allowing hawks to fly the way they do. For sure, hawks do not fly based on an understanding of the properties of logarithmic spiral. How can they show the corresponding behavior? The often invoked evolutionary explanation offers poor help: it accounts for behavior in terms of its reproductive advantage. As the spiral-like flight proved advantageous for hawks, those who performed it were able to generate more offspring, while the others extinguished. No generative theory here: it tells us not how hawks produce the behavior in question. We could use the mathematical theory to describe their behavior, and incorporate the mathematical explanation into a set of *ad-hoc* behavioral rules for reproducing it. But neither the mathematical explanation, which describes internal causes, nor the set of *ad-hoc* rules are generative.

Now, a fully generative explanation implies a more general theory of how external causes, including fitness-enhancing effects, get converted into internal reasons (what sometimes are called proximate causes of behavior). Agent-based models are often limited in focus, and not easily compatible with the temporal perspective and the theoretical requirements of a fully generative - in the sense here intended - explanation. Do we always need a generative explanation? Not really, as *ad-hoc* rules sometimes are just all that is needed to explain behavior. This is the case of entirely programmed organisms, and it may even be the case of hawks, as far as we know. Sometimes, instead, you need more. Suppose you want a hawk to learn a new trick with respect to the approach behavior. That is, you, or nature, in the form of new environment - perhaps, but we're letting imagination run wild here, in the form of a prey that develops a counterstrategy to the spiral. Then, immediately how the flight is generated becomes important: how much learned, how much hard wired, and where; in a plastic neuronal connection, or in a fixed relative placement of eye and bone? Suddenly, to reproduce their behavior you would need more than a rigid set of rules; you need to know how it is generated.

Cognitive modeling aims at finding the general mechanisms yielding the wide spectrum of behaviors of relatively autonomous systems. Of course, you don't need such mechanisms to simply reproduce behavior. The more specific the target behavior, the lesser you need a cognitive agent-based model. Since ABM is often used to investigate fairly specific phenomena, either mathematical model or a set of *ad-hoc* rule are preferred over cognitive modeling. But together with cognitive modeling, we also dispense away with truly generative modeling.

### 2.4. Why bother with generative explanation

One might say, who cares after all? Provided we can reproduce behavior, observe it and make artificial experiments to optimize it, why bother with theory-driven generative modeling? There are several reasons. One is that a truly generative explanation is needed to model complex social dynamics. For universal admission, the dynamics of social entities and phenomena is at least bidirectional if not multidirectional. Entities and properties emerge from the bottom up and retro-act on the systems that have generated them. Current agent-based models instead simulate only emergent properties, i.e., the way up of social dynamics. To mention only a few examples, the ABM literature offers countless models of the emergence of segregation, norms, reciprocity, altruism, cooperation, punishment, conventions, institutions, coalitions, leadership, hierarchies, the modern state. Studies of different types and levels of downward causation are much less frequent (to cite some exceptions, see Gilbert, 2002; Conte et al., 2013). However, how to change self- and other-damaging behaviors (i.e., smoking, over-eating, etc.) was ranked as the fourth most important among the top-ten hard problems the social sciences will have to address in the near future (Giles, 2011).

Agents should not be taken for granted as they change under different types and degrees of social influence. Entities at the macroscopic level affect them and their behavior, and we must understand how this can happen if we want to drive, enforce, or prevent such an influence. This a line of research that presents obvious ethical issues, but at the same time addresses themes so important that social science cannot just leave them alone, or, even worse, desert them to market solutions. For example, at least in some fields, we badly need to know how to reduce or control people's overconfidence, for example in finance, where it so heavily contributed to the last financial crisis (see Akerlof and Shiller, 2010), causing a disruption of global scale; how to change people's bad food habits, which are mainly responsible for highly diffuse diseases as diabetes; how to make low compliant populations to obey the norms, how to increase social trust, reduce hostility toward out-groups, favor communitarian attitudes, and so on. All of these questions might find useful answers based on reality mining. Through Google or Yahoo we may trace people's habits, moods, investment decisions, political views, risk propensity and attitudes toward culture, education and migration. Based on this information, we may drive production, capital movements, business strategies, political decisions, and international cooperation. But we will not be able to suggest effective plans for modifying such behaviors and the underlying mental states, unless we understand the mental dynamics and how this interacts with the social dynamics, and model the cognitive mechanisms that respond to external influence and rule behavioral change. In absence of such theory and model, we will not get to the core of hard problems.

### 2.5. A missed opportunity

ABM is a powerful means for investigating the hinge between different domains of reality, including economy, environment, and society: systems' behavior at different levels of scale. It is necessary to explain phenomena pertaining to any domain of reality that is heavily dependent on the behavior of autonomously interactive systems, as was convincingly argued by Epstein. More, ABM is unique for allowing a generative approach to behavioral systems in the sense here defined, and somewhat different from Epstein's, i.e., to describe phenomena in terms of the external and internal mechanisms that produce them.

However, ABM seems to have fulfilled its mission only in part. Its generative capacity has been deployed to a lesser extent than could have been the case. The practice of ABM missed the opportunity it provided: paradoxically, the same principle that led it to a fast popularity, like the KISS principle—i.e., keep it simple, stupid - introduced by Axelrod (1997), and moreover the procedure to find the minimal required conditions to obtain a given phenomenon, do now sentence ABM to a premature end. The KISS principle still drives most of the simulation work: we have performed a check on a whole year of JASSS, a journal that we consider representative of the files. In 2013, JASSS published 49 papers, of which 38 could be classified as simulations (the rest is composed mostly by theoretical papers). Of those, 30 could be considered as following the KISS advice, which makes about the 80% of published papers.

If internal mechanisms are *ad-hoc* and arbitrary, why don't dispense away with them in favor of more powerful quantitative modeling allowing the same phenomena to be accurately predicted? Why bother with agents, if one can apply computational tools to reality mining and platforms to large scale real-world data-driven simulations, and aim at even higher orders of magnitude, enabling us to forecast events at aggregate levels, such as epidemics, climate change, and traffic jams? Couldn't it be the case that a mere quantitative use of computational tools be enough to forecast financial crises, social instability, and even human well-being?

It could be the case, indeed. However, centuries of failed attempts (see the “Social Physics” case mentioned above) make us doubtful. But what is maybe more important, by pursuing this quantitative approach alone, science would have lost a wonderful opportunity: to understand the micro-foundations of phenomena at aggregate levels and how the latter (re)generate them.

## 3. Computational social science

Science, like history, is not a linear process. A decade ago, social, and behavioral science dropped the disciplinary label (Conte, 2002) under the influence of an entirely new field, ABM. In the last couple of years a CSS is being re-proposed. But CSS is being practiced since a couple of decades if not earlier. What is new to the current program?

Computational Social Science (from now on CSS) can be meant in at least three different ways, the deductive, the generative, and the complex one; and it should be made clear which one we are referring to. As these are conceptual, rather than empirical, variants, there is no need to have each of them matching a defined historical example of CSS, since concrete examples are often a mix. Let us characterize variants also with reference to existing programs and try to forecast what their consequences might be.

### 3.1. The deductive variant

The second half of the last century is constellated of attempts to apply the theory-building instruments of mathematics and the theory-testing tools of computer science on one side, game theoretic, and logic-based computational models on the other, to describe and explain social phenomena. The latter, in particular, attempted at deducing properties at the macro-level from general assumptions at the micro-level. Expectations á la *homo economicus*, allowed by the theoretical framework, turned out to be wrong, what did not imply that the approach was incorrect, only that it had been based on the wrong assumption, depending on the theoretical and sometimes ideological positions of the authors. What was worse, these position were often left implicit. The deductive variant consists of formulating the mathematical equations that account for the phenomena to be explained. With the support of observation and data gathering, parameters can be assigned their correct values. Although the theoretical framework is often much too simple, the general program scarcely interdisciplinary, and the ambition for social impact mainly based on a rather prescriptive view of micro-level theory, deductive CSS yielded a foundational, general, explanatory theory of social systems. A lesson we should not forget.

### 3.2. The generative variant

The decline of the rationality paradigm produced several consequences. One of these was a stronger and more interdisciplinary effort to ground computational models on explicit models of the micro-foundations. This led to the advent of the generative variant of ABM, which derives its explanatory vocation and micro-foundational framework from the deductive variant. Unlike it, generative science aims at modeling operational microscopic rules that generate macroscopic phenomena, rather than formulating mathematical equations from which to deduce them. The explanatory vocation is declined in a radically different way: rather than describing a causal process from the outside, the modeler attempts to show the internal rules that initialize it and follow the unfolding of it all the way up to the observed effects.

As argued in the preceding section, however, ABM fulfilled its mission, provide generative theories, to a lesser extent than was expected. If the deductive variant was found to theorize upon fairly abstract phenomena and has often been criticized for its poor predictive capacity, the generative variant did not prove any better at prediction, partly due to problems of validation and calibration.

### 3.3. The complex variant

Inductive computational science is certainly not new (Newell and Simon, 1976). The necessity to combine mathematics and logic with learning, probability, and induction is receiving a growing attention since the early nineties in several computational disciplines such as knowledge representation, reasoning about uncertainty, data mining, and machine learning. Nor is new the use of computational instruments for quantitative social science: it suffices to think of the wide application of statistics package for social scientific research, and by the number of repositories and archives of social scientific data (for example, http://www.data-archive.ac.uk/). However, techniques of data-mining are exercising an even stronger influence on the social sciences. The use of advanced computer technology by social scientists is also shown by sites where freely available web resources are assembled with information on how access social scientific data (see for example, http://guides.lib.wayne.edu/socialsciencesdata), and by funded programs for interfaces between computer and social sciences.

A new impulse to computer-based quantitative social science is coming from the science of complexity, which is now going through a season of deserved popularity. The use of complex systems' methods, models and techniques to economic systems goes back to the nineties (for a rather informative introduction, see Mantegna and Stanley, 2000), and the welcome received by mechanical statistics in the field of economics and finance was such as to encourage its wider application to the rest of the social sciences. The popularity of sociophysics grew even more under the influence of success stories, especially concerning the domain of pedestrians' crowd (Helbing et al., 2000) and that of epidemics (Pastor-Satorras and Vespignani, 2001). In the last few years, a diffuse uncertainty related to globalization, international and cultural conflicts, and the recent financial crisis, led to the necessity to anticipate and manage critical events on the front-stage. Not only stakeholders and policy makers but also, and consequently, research and development funding agencies and evaluators laid emphasis on science as a system of warning, a source of anticipatory information on the performance of aggregate systems, simultaneously triggering and guiding the action of politicians, administrators, and businessmen. But science is more than anticipation. It is first of all explanation. Accurate prediction can do without explaining, especially if it is based on large datasets and sophisticated techniques for extracting knowledge out of them. Science cannot. Of course, explanation may be allowed by statistical analysis. For example, topological properties of complex networks are found among the main factors affecting epidemic dynamics (for a review, see Yang et al., 2007). But this is not always the case. Indeed, this is not the paradigmatic case in those social phenomena in which behavior can be assumed to be irrelevant, or non-influential.

Behavior is irrelevant or non-influential in social dynamics where the implications of the phenomenon in question are social, but its nature is not. To go back to epidemics, the nature of epidemics is biological. The level of reality involved entities belong to does not matter for the observed phenomenon to take place: the nature of entities involved in and target of epidemics matters not. In the spread of epidemics, the difference between human behavior and that of particles in the space does not matter, nor does the difference from carriers and the viruses they carry around. But in other cases, that is, when the nature of behavior matters, accurate statistical analyses of social dynamics can maybe reach predictive power but cannot fully explain what is going on.

As a hypothetical example, suppose we want to know what are the main factors responsible for the dynamics of opinions. Again, current models (Deffuant et al., 2001; Galam, 2002; Hegselmann and Krause, 2002; Castellano et al., 2009), find that the structure of the network of communication affects opinion dynamics. Of course, the source of information also matters; a contrasting source may inhibit the effect of media broadcasting and the process is non-linear: under a given critical level of coverage, the broadcasting message may be inhibited by a “contrarian” opinion spread through word of mouth. Analogously, below a critical level of confidence “contrarian” opinions may reach all agents (Castellano et al., 2009). Social dynamics are often non-linear and typically smolder at some length under the ashes and only subsequently surface in convergent opinions or behaviors. Suppose one predicts the moment(s) at which this will happen in real-world dynamics thanks to statistical analysis and physical models. The question is why it happens. Of course behavior is irrelevant to predict when convergence will occur. But it matters if, for example, we want to affect the process, by shortening or delaying it, or even prevent it; to educate people to a higher autonomy; to favor info-diversity; finally, to convert opinions in something more solid and resistant, like knowledge, and so on.

People withdrawing support from political leaders is a good example of non-linear opinion dynamics. It is unclear when people change their minds and turn down their leaders. The destiny of a popular (and often populist) figure is often decided upon in a very short time. Today, those who enjoyed the favor of their followers until yesterday, may suddenly lose popularity and fall in disgrace, what is again a matter of threshold: after a certain level of spreading, and perceived spreading, agents are led to modify their opinions, what probably reveals an interesting effect of shared representations about shared opinions on one's confidence level. Possibly, such a lowering confidence leads agents to be more eager to change opinions.

However, the circuit may be completely different: agents may resist pressure to change opinions despite contrasting evidence for reasons of cognitive dissonance. The more the contrasting evidence they gather, the higher the dissonance. To reduce it, they try to ignore evidence that is less costly than change opinion, which imply dropping the previous commitment and making a new one. As the perceived distance from others' opinions increases, however, agents either hide their opinions or must defend them openly. If they choose the latter strategy, they may even end up by accepting to form part of a minority. If they take the former option they cannot get along with deception too long as cognitive dissonance increases. Consequently, they accept others' opinions as own, and are likelier to convert them in open behaviors to convince others and themselves about the solidity of their new opinions. Both routes imply critical thresholds for totally different reasons. To act efficaciously on this process, we must be clear what is actually going on. Confidence has different implications from cognitive dissonance and self-deception. To increase confidence may lead to higher stability in the former case, but not in the latter.

To sum up, to model social dynamics without taking into account the internal (cognitive) dynamics of the entities involved in a social phenomenon does not prevent accurate predictions of critical events and changes. It may even allow to find out factors responsible for such events and changes, and this is the case with dynamics for which the social nature of behavior is irrelevant. To understand internal dynamics is crucial instead whenever we need not only to anticipate but also to understand events for which behavior is relevant. Model the internal dynamics of events is necessary not only for scientific reasons but also for guiding intervention.

## 4. Toward a new interdisciplinary foundation for CSS

The program for CSS needs clarification. Why would such a program be necessary, if we practice CSS since at least a couple of decades? Of course one might say that we need to introduce a new Curriculum at the academic level, and that to do this implies to form a new, cohesive, scientific community, form associations, give visibility to this new Curriculum, strengthen the academic, editorial, and political power of the underlying community etc. However, the reason for a program on CSS is not only political but also scientific. As seen so far, there are different variants of CSS and to take a pluralistic approach to it may be considered wise. CSS could be seen today as a larger umbrella under which different approaches might coexist and somehow feel legitimate. Hence, generative ABM might be practiced by a subset of social scientists, while others might prefer a purely quantitative approach, based on data-mining and numerical simulation, and still others might continue to formulate abstract theories of social action in elegant equations and deduce their macro-level consequences.

The main thesis of this paper is that such a *multidisciplinary* program for CSS would be another lost occasion for science. It would but result either in tight but essentially useless theories, or in accurate predictions of poorly understood social phenomena. In the best possible case, mathematicians will go on citing one another in fairly close circuits of beautiful minds, physicists will find new phenomena affected by the properties of scale-free networks, and social scientists will give up generative ABM in the desperate attempt to produce competitive quantitative social science and get reasonably high scientific scores.

An interesting, innovative program in CSS can only be *interdisciplinary*. Why and where is the difference? The reason lies in the necessity to take advantage from the different modeling methods and techniques to both understand and predict the same phenomena! The difference of interdisciplinary from multidisciplinary CSS consists not only of a convergent investigation of the same phenomena from different perspectives and involving different competencies, what would already be a step ahead with regard to current practice, but in a more radical process aimed at multilevel and modular modeling. Such a type of modeling would allow to describe the dynamics of given phenomena at aggregate levels based on large datasets, find out the criticalities thanks to complex dynamic systems models, make hypotheses about the behavioral dynamics when this is relevant, use ABM to check internal consistency and observe the resulting states at the aggregate level, apply cross-methodological experimental methods to validate the hypotheses against real-world data, update data-mining methods, models, and probability distribution models to newly acquired knowledge, and use mathematical equations when possible to close the number of states resulting at the aggregate level.

An interdisciplinary endeavor like this certainly points out some new challenges: not only to extract knowledge from larger and larger datasets, not only develop simulators that scale up of several orders of magnitude, or feed simulation and data-mining with online real-data, not only to develop supercomputing infrastructures and systems to transfer data to supercomputing platforms, but also develop simulation platforms that scale up both in terms of systems' dimensions and in terms of levels of complexity. We need to account for large-scale systems as well as more complex entities. We need to apply simulation methods to understand the social and the mental dynamics and to describe their interrelationships. Last, but not least, we need incentives that are compatible with such an endeavor—publication-wise and career-wise. This is a challenge for a program on CSS that deserves attention and investment. CSS ought to accept it, or another occasion will be lost for founding a novel, integrated, interdisciplinary, falsifiable science of society helping us to solve transformative and foundational problems.

## Author contributions

Rosaria Conte and Mario Paolucci elaborated together the ideas presented in the paper. Rosaria Conte drafted the first version. Mario Paolucci substantially revised it for important intellectual content.

## Funding

This paper benefits from the stimulating discussions in the *FuturICT* FET Flagship Pilot Project (EU FP7/2007-2013, g.a. no. 284709). The publication of this work is funded by the PRISMA project (PON04a2 A), within the Italian National Program for Research and Innovation (Programma Operativo Nazionale Ricerca e Competitività 2007–2013).

### Conflict of interest statement

The authors declare that the research was conducted in the absence of any commercial or financial relationships that could be construed as a potential conflict of interest.
